# Supplementary figures and images for: Heatwaves and dengue outbreaks in Hanoi, Vietnam: New evidence on early warning
Source: PLoS Negl Trop Dis. 2020 Jan 21;14(1):e0007997. doi: 10.1371/journal.pntd.0007997 (PMC6994101; doi:10.1371/journal.pntd.0007997)

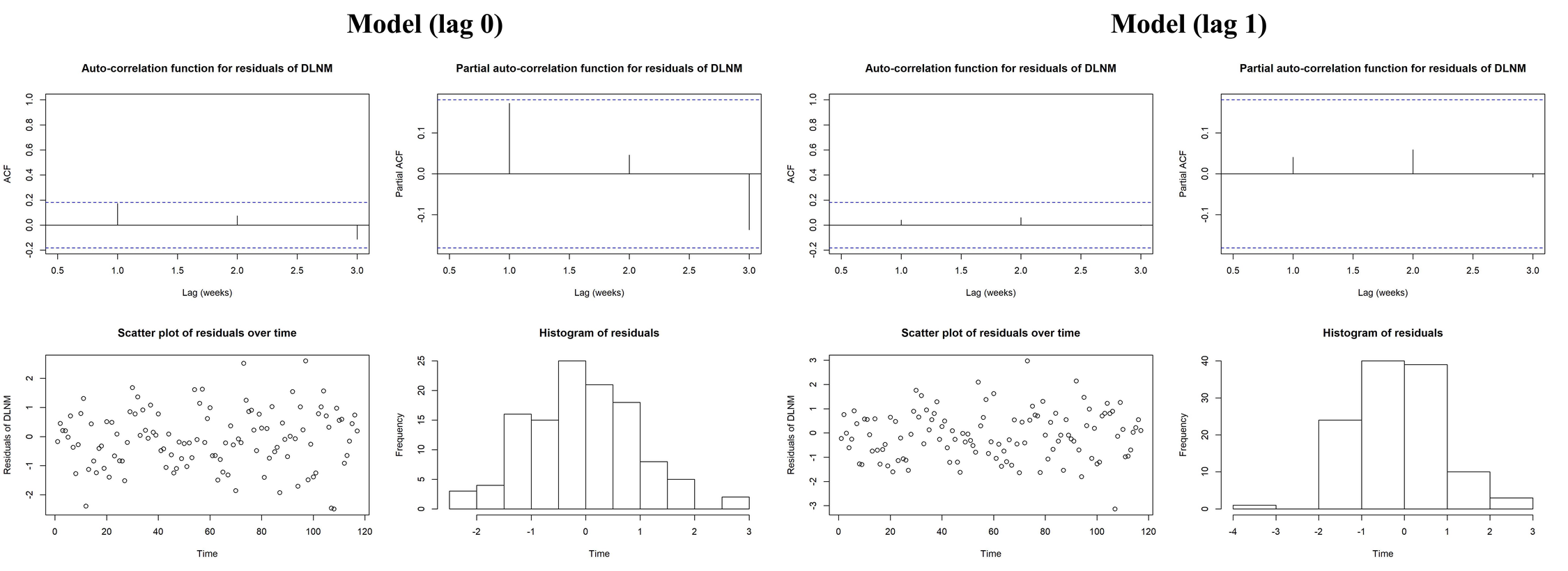

Supplement: S1 Fig — (TIF) [file pntd.0007997.s001.tif]

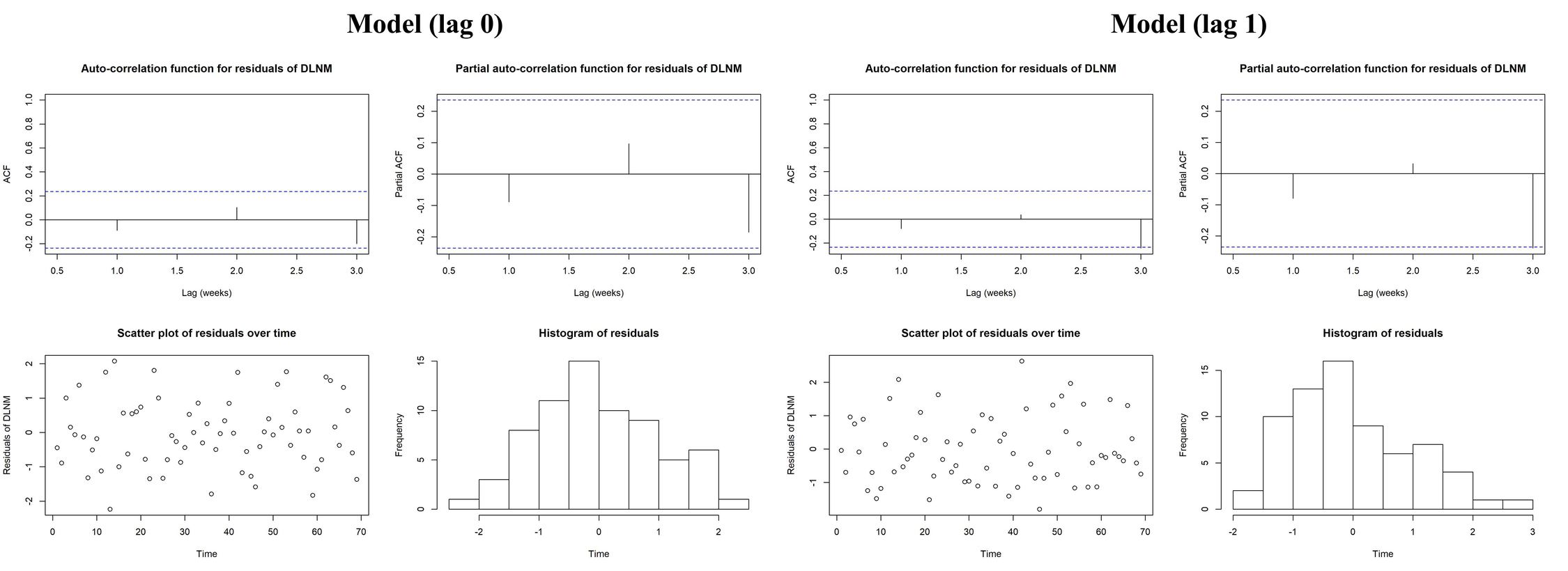

Supplement: S2 Fig — (TIF) [file pntd.0007997.s002.tif]

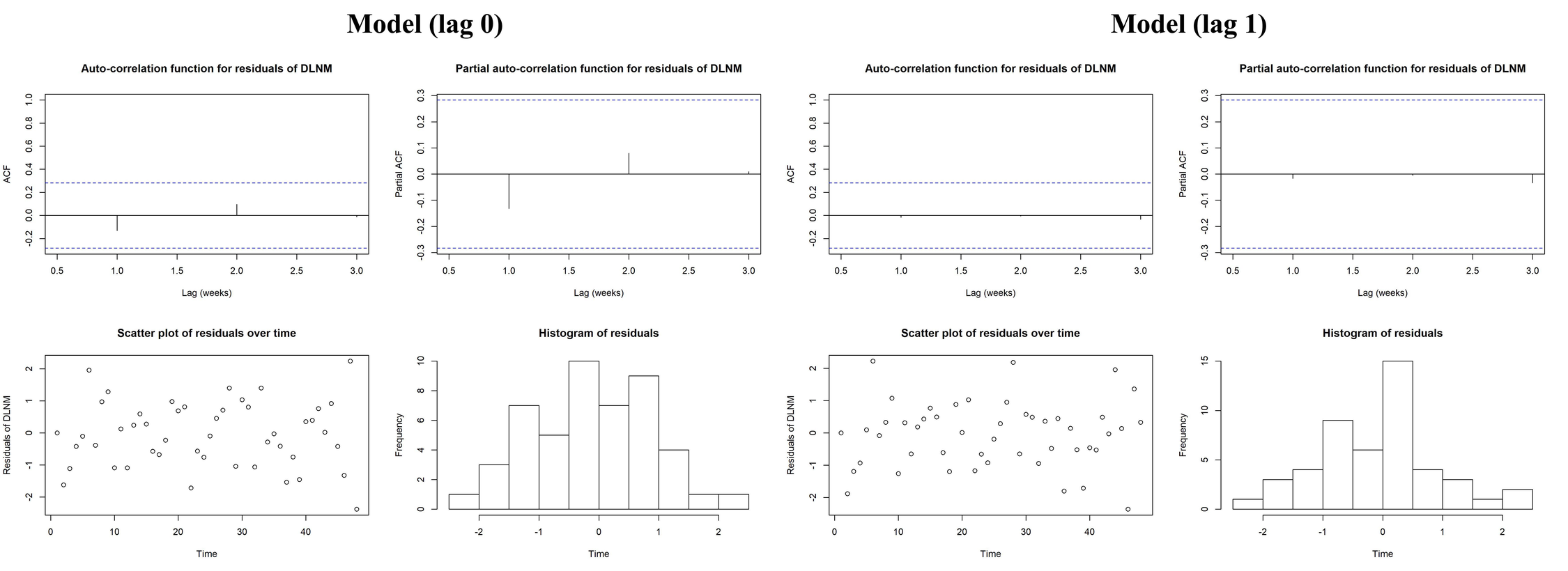

Supplement: S3 Fig — (TIF) [file pntd.0007997.s003.tif]

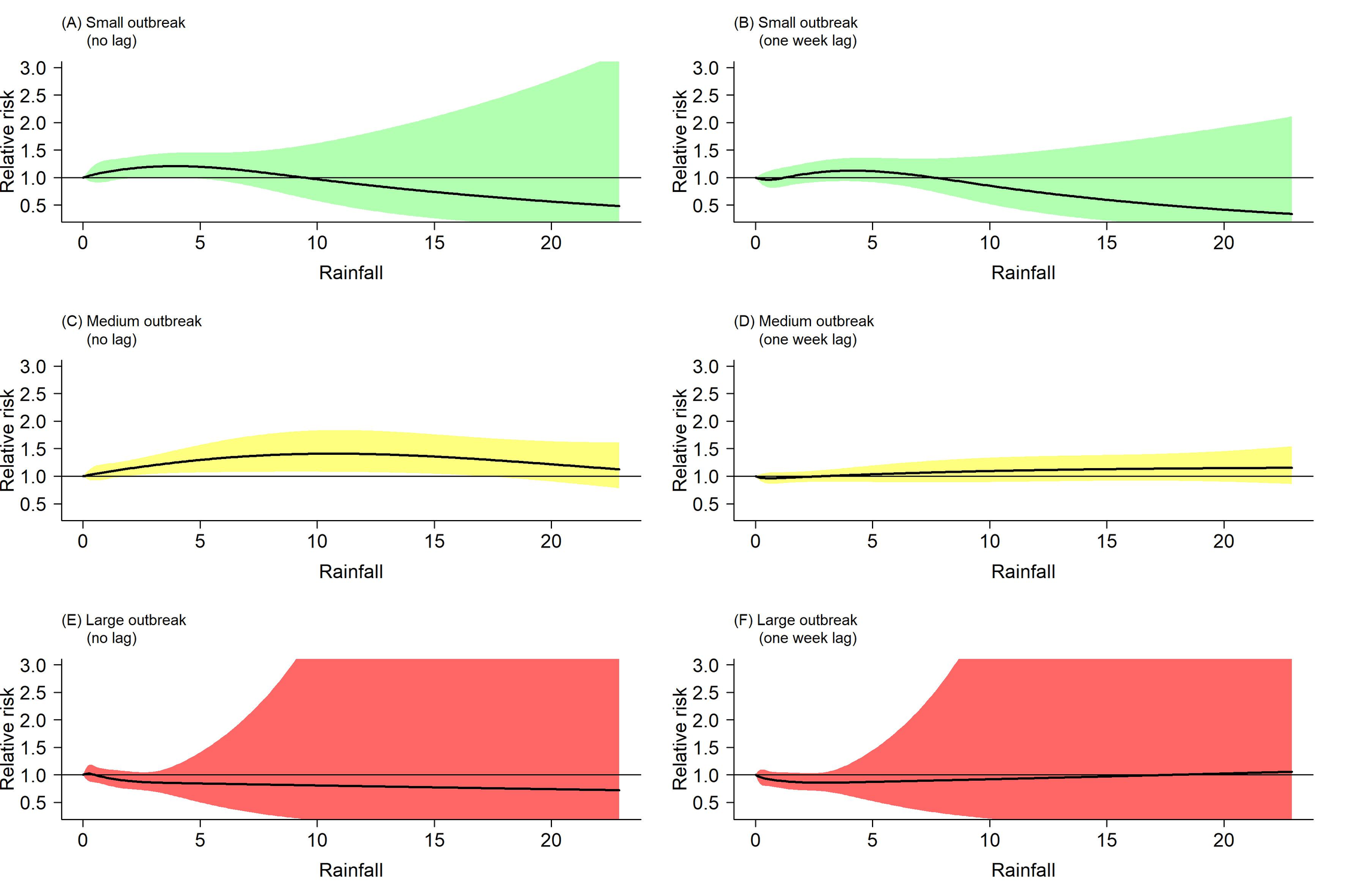

Supplement: S4 Fig — Black lines indicate the relative risk and the shaded area the 95% confidence interval; dotted lines are the threshold temperature with the highest relative risk. (TIF) [file pntd.0007997.s004.tif]

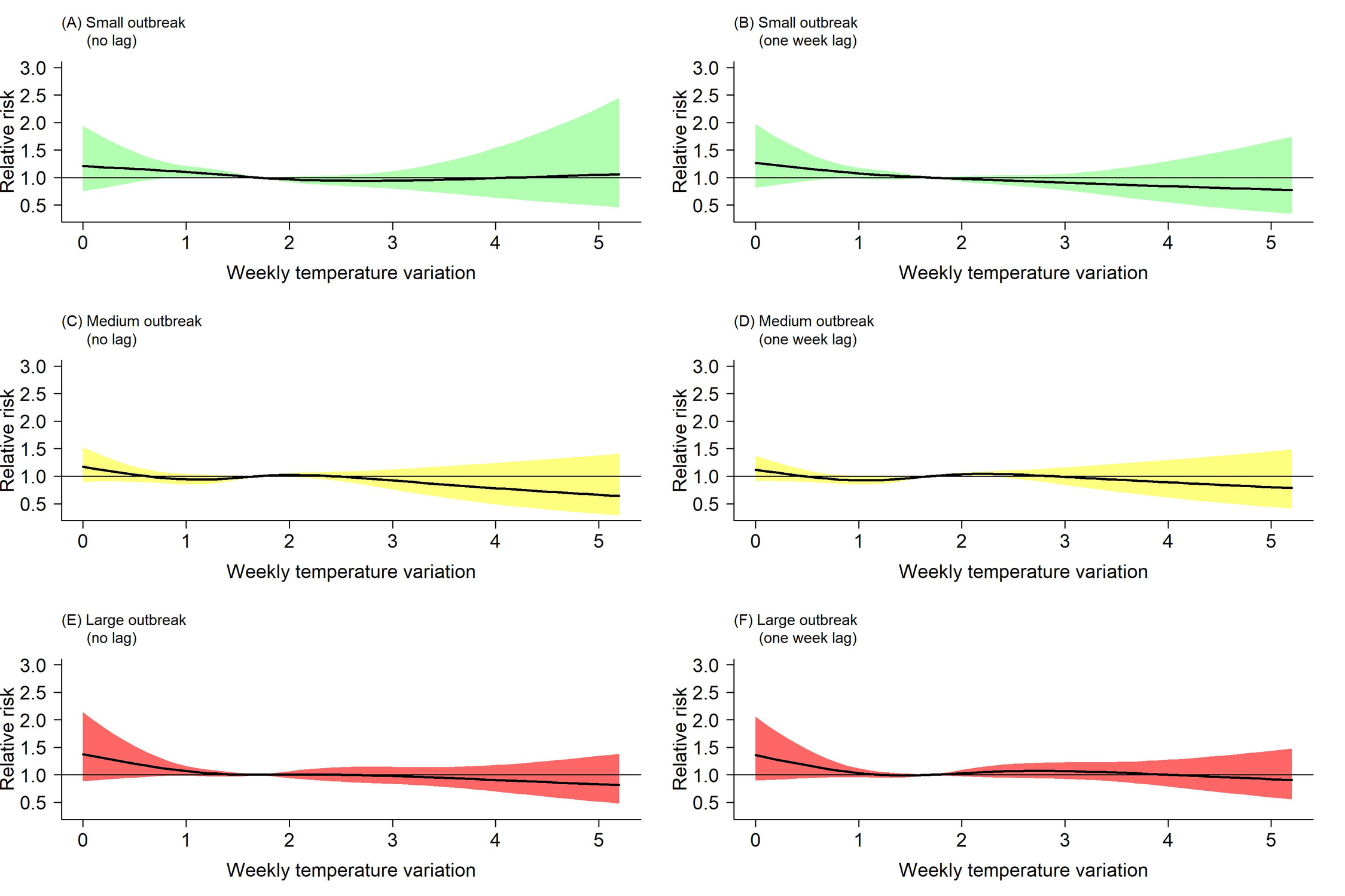

Supplement: S5 Fig — Black lines indicate the relative risk and the shaded area the 95% confidence interval; dotted lines are the threshold temperature with the highest relative risk. (TIF) [file pntd.0007997.s005.tif]

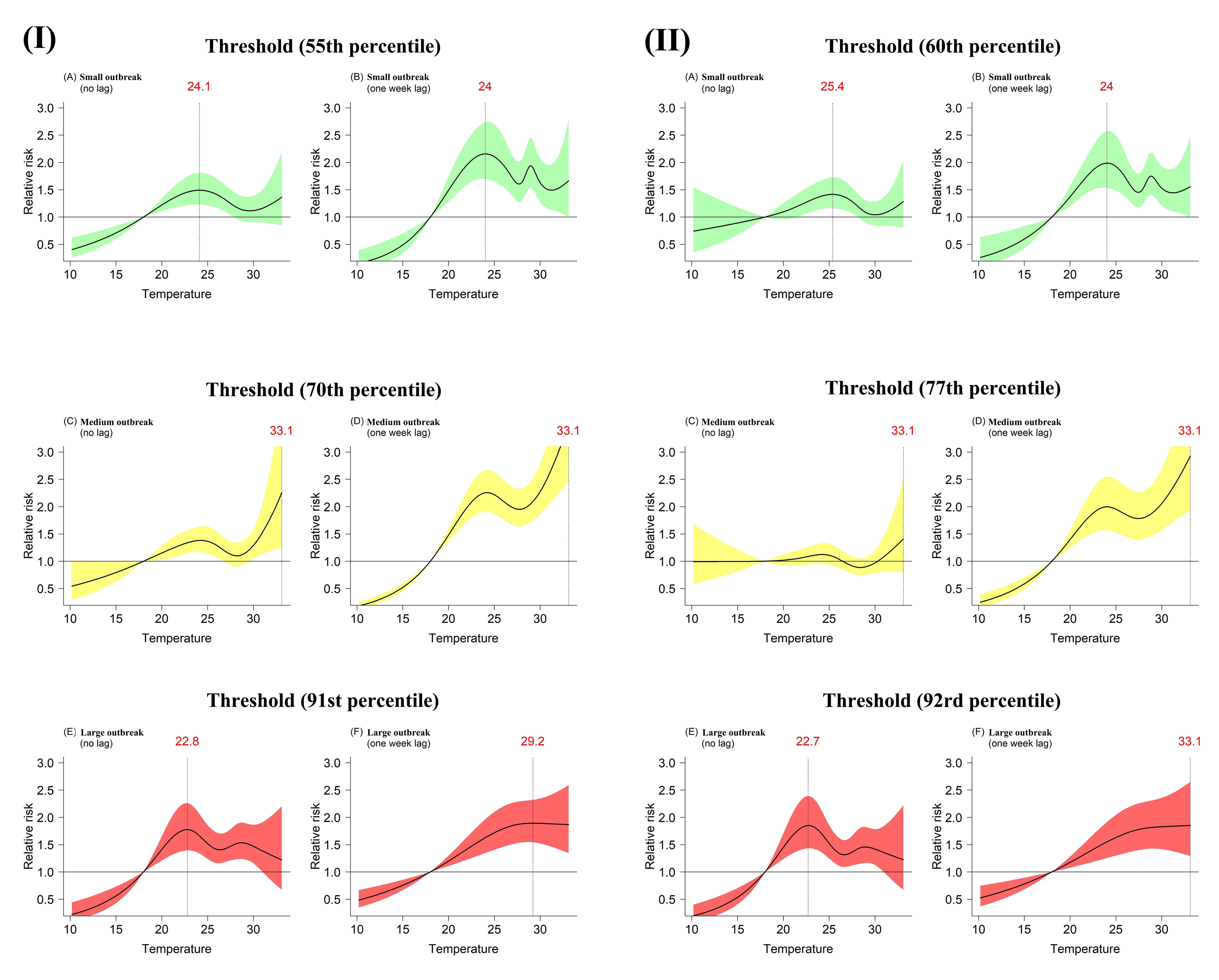

Supplement: S6 Fig — (TIF) [file pntd.0007997.s006.tif]

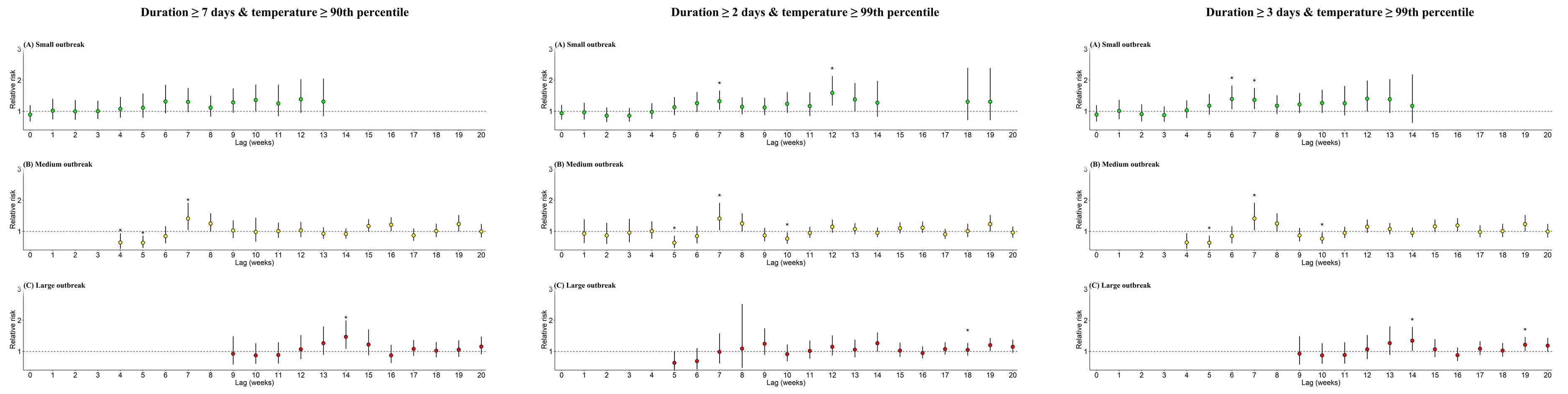

Supplement: S7 Fig — y-axis represents the relative risk; x-axis represents the lag weeks from 0 to 20; Three heatwave definitions were used including: (1) seven or more days with temperature exceeding 90th percentile of daily temperature distribution; (2) two or more days with temperature exceeding 99th percentile of daily temperature distribution; and (3) three or more days with temperature exceeding 99th percentile of daily temperature distribution. (TIF) [file pntd.0007997.s007.tif]
