# Supplementary material for: Heatwaves and dengue outbreaks in Hanoi, Vietnam: New evidence on early warning
Source: PLoS Negl Trop Dis. 2020 Jan 21;14(1):e0007997. doi: 10.1371/journal.pntd.0007997 (PMC6994101; doi:10.1371/journal.pntd.0007997)
Supplement: Attachment — Submitted filename: Cheng et al.docx [file pntd.0007997.s008.docx]

Cheng et al. Dengue and heatwaves

Interesting paper and quite surprised no one has looked at this. Overall, I like this paper very much. It is very clear and well written and shows important results. It could do with some clarification in parts as outlined below.

1. To note, there is a relatively recent paper on mosquito dynamics and heatwaves that is worth looking at.

Potential effects of heat waves on the population dynamics of the dengue mosquito Aedes albopictus. PLoS Negl Trop Dis. 2019 Jul; 13(7): e0007528. Published online 2019 Jul 5. doi: 10.1371/journal.pntd.0007528 PMCID: PMC6645582 PMID: 31276467

**Statistics**

Just some clarification

2. Can the authors clarify whether the model used was a GAM (or other) for the non-linear analyses? And was non-linearity also considered for rainfall?

3. In place of diurnal temperature range the authors fitted weekly temperature range (WTR). Maybe clearly state this as DTR has a well-known effect. I’m not sure if anyone has compared WTR with DTR.

4. The spatial scale: District was not included as a random factor?

5. The choice of 1 week lag. Were other lags considered, as different lags have been previously found for both rainfall and temperature.

**Results**

6. It might be helpful in a line or two just to say what the effects of rainfall and temperature range were.

7. Figure 2 shows the relationship with temperature after taking into account all the other confounding covariates. Correct?

8. Figure 5. It’s quite hard to read and see the significance levels. I’m quite surprised that any of those are significant. All the pairs look very similar. Here the weekly number of cases is the unadjusted (for other variables) value?

9. The effect of heatwaves: if indeed there is a true effect, which is remarkable to detect with only two heatwave years, perhaps some mention should be made of the serotypes involved to allay fears that this is not confounded by impact of herd immunity.

**Discussion**

10. Line 345 “So far, few studies have assessed dengue burden arising from the short-term temperature effects.” There have been a lot of studies on this or perhaps I am misunderstanding what the authors mean. Would also be good to cite some references here.

11. Maybe insert some comment about serotypes as a limitation, unless this information is available.

12. The association of heatwaves and increased outbreaks may well reflect the impact of heatwaves on human health generally. There’s quite a lot on this in non-infectious diseases. Heatwaves make people sick and effect the immune response.
