# Supplementary material for: Heatwaves and dengue outbreaks in Hanoi, Vietnam: New evidence on early warning
Source: PLoS Negl Trop Dis. 2020 Jan 21;14(1):e0007997. doi: 10.1371/journal.pntd.0007997 (PMC6994101; doi:10.1371/journal.pntd.0007997)
Supplement: Attachment — Submitted filename: Response to reviewers comments.docx [file pntd.0007997.s009.docx]

**Point-by-point responses to the reviewers’ comments:**

We thank all reviewers for their positive and constructive comments, which are valuable in improving the quality of our manuscript.

Relevant changes in the revised manuscript have been marked red according to reviewers’ comments.

**Reviewer #1:**

Interesting paper and quite surprised no one has looked at this. Overall, I like this paper very much. It is very clear and well written and shows important results. It could do with some clarification in parts as outlined below.

1. To note, there is a relatively recent paper on mosquito dynamics and heatwaves that is worth looking at.

Potential effects of heat waves on the population dynamics of the dengue mosquito Aedes albopictus. PLoS Negl Trop Dis. 2019 Jul; 13(7): e0007528. Published online 2019 Jul 5. doi: 10.1371/journal.pntd.0007528 PMCID: PMC6645582 PMID: 31276467

**Response:** Thanks for your suggestion. The above-mentioned paper is an important research on heatwaves and mosquito dynamics. We have cited the paper in our revised manuscript (lines 117-118, page 5; lines 371-372 & 393, page 19).

**Statistics**

Just some clarification

2. Can the authors clarify whether the model used was a GAM (or other) for the non-linear analyses? And was non-linearity also considered for rainfall?

**Response:** The model we used for the data analysis was a generalized linear model (GLM) with a negative binomial link. This model was constructed within the framework of distributed lag non-linear model. Similar modelling approach has also been used in many previous studies to examine the non-linear relationship between environmental exposures and health outcomes (Lowe et al., 2018; Cheng et al., 2018; Xu et al., 2014). In the revised manuscript, we have clarified the model used for the data analysis (lines 185-186, page 8).

For the non-linear relationship between rainfall and dengue, we used a natural cubic spline smoothing function with three degrees of freedom, which has been mentioned in the initial manuscript (lines 189-190, page 8).

References:

Cheng J, Zhang Y, Zhang W, et al., 2018. Assessment of heat- and cold-related emergency department visits in cities of China and Australia: Population vulnerability and attributable burden. Environ Res. 166:610-619. doi: 10.1016/j.envres.2018.06.026.

Lowe R, Gasparrini A, Van Meerbeeck CJ, et al. 2018. Nonlinear and delayed impacts of climate on dengue risk in Barbados: A modelling study. PLoS Med. 15(7):e1002613. doi: 10.1371/journal.pmed.1002613.

Xu HY, Fu X, Lee LK, et al., 2014. Statistical modeling reveals the effect of absolute humidity on dengue in Singapore. PLoS Negl Trop Dis. 8(5):e2805. doi: 10.1371/journal.pntd.0002805.

3. In place of diurnal temperature range the authors fitted weekly temperature range (WTR). Maybe clearly state this as DTR has a well-known effect. I’m not sure if anyone has compared WTR with DTR.

**Response:** Thanks for your comments. The motivation of including weekly temperature range (WTR) in the model is because previous studies have found association between diurnal temperature range (DTR) and dengue incidence (Sharmin et al., 2015). This reason has now been added to the revised manuscript (lines 186-187, page 8).

To our knowledge, no study has compared effects of WTR and DTR. This topic will be further studied in our upcoming work. Thanks.

Reference

Sharmin S, Glass K, Viennet E, Harley D. 2015. Interaction of Mean Temperature and Daily Fluctuation Influences Dengue Incidence in Dhaka, Bangladesh. PLoS Negl Trop Dis. 9(7):e0003901. doi: 10.1371/journal.pntd.0003901.

4. The spatial scale: District was not included as a random factor?

**Response:** This study is purely a time series analysis. The data analysis of this study was conducted at the city level, which has more powerful sample size to ensure the statistical power. So, we did not consider the data analysis at finer spatial resolution and the district was not included as a random factor in the model.

Similarly, to investigate the association between temperature and dengue, the city-level time-series analysis has been widely conducted in many previous studies (Banu et al., 2014; Wu et al., 2018).

References:

Banu S, Hu W, Guo Y, Hurst C, Tong S. 2014. Projecting the impact of climate change on dengue transmission in Dhaka, Bangladesh. Environment international. 63:137-42.

Wu X, Lang L, Ma W, Song T, Kang M, He J, et al. 2018. Non-linear effects of mean temperature and relative humidity on dengue incidence in Guangzhou, China. The Science of the total environment. 628-629:766-71.

5. The choice of 1 week lag. Were other lags considered, as different lags have been previously found for both rainfall and temperature.

**Response:** Yes, we considered the temperature effects at two different lag windows: lag 0 week (within the same week of temperature change) and lag 1 week (one week after temperature change), which is in line with a recent study (Wu et al., 2018). In our study, we found that temperature at lag of 1 week has the highest correlation with dengue fever. However, to avoid the multicollinearity issue of model among the temperatures at different lags, we only selected lag 0 and lag 1 week in our model.

References:

Wu X, Lang L, Ma W, Song T, Kang M, He J, et al. 2018. Non-linear effects of mean temperature and relative humidity on dengue incidence in Guangzhou, China. The Science of the total environment. 628-629:766-71.

**Results**

6. It might be helpful in a line or two just to say what the effects of rainfall and temperature range were.

**Response:** Regarding the effects of rainfall and temperature range, we did not observe the association of dengue risk with rainfall and weekly temperature variation, except for rainfall increases within the first week that were associated with higher dengue risk during medium outbreaks (S4-S5 Figs). This finding has been added to the revised manuscript (lines 255-257, page 12).

7. Figure 2 shows the relationship with temperature after taking into account all the other confounding covariates. Correct?

**Response:** Yes. Figure 2 was plotted after controlling for potential confounding covariates including weekly temperature variation, weekly average rainfall, long-term trend, an auto-regressive term, and each year's population in log scale. The confounders included in the model have been added to the caption of Figure 2.

8. Figure 5. It’s quite hard to read and see the significance levels. I’m quite surprised that any of those are significant. All the pairs look very similar. Here the weekly number of cases is the unadjusted (for other variables) value?

Response: Figure 5 has been re-plotted with larger and clearer text and labels. This figure is now much easier to read and see the significance levels (page 16).

In this Figure, three pairs are not statistically significant, as indicated by the "ns" sign.

The weekly number of cases in this Figure is the observed number of cases, rather than the number obtained from the model that adjusted for other variables. We compared the weekly number of cases in years with and without heatwaves, and also considered the delayed effects of heatwave (detailed in the method section, lines 217-223, pages 9-10).

Since this study is a preliminary analysis to explore the impacts of heatwaves on the magnitude of dengue outbreaks, more work will be conducted to further prove our findings.

9. The effect of heatwaves: if indeed there is a true effect, which is remarkable to detect with only two heatwave years, perhaps some mention should be made of the serotypes involved to allay fears that this is not confounded by impact of herd immunity.

Response: Thanks for your suggestion. The heatwave event identified just in two years was based on one heatwave definition, namely seven or more days with temperature exceeding 95th percentile of daily temperature distribution (Fig. 1C). Besides, three other heatwave definitions were also used in this study, with heatwave event being observed in many years such as the year 2009, 2010, 2013, 2015, and 2016 (Fig. 1B). Importantly, the findings about the effects of heatwaves were relatively robust across different heatwave definitions (S7 Figs). These findings consistently suggest that heatwaves could have a significantly impact on dengue.

Admittedly, the impact of herd immunity may be a potential factor that influences the results on heatwave effects. But the information about serotypes of dengue is not available in this study. This limitation has been highlighted in the revised manuscript (lines 429-431, page 21).

**Discussion**

10. Line 345 “So far, few studies have assessed dengue burden arising from the short-term temperature effects.” There have been a lot of studies on this or perhaps I am misunderstanding what the authors mean. Would also be good to cite some references here.

Response: Thanks for your comment. Although many studies have been performed to examine the association between short-term temperature and dengue, most of which used the relative risk or odds ratio to assess the short-term temperature effects (Fan et al., 2014; Wu et al., 2018). So far, very few studies have evaluated the dengue burden attributable to temperature, measured as the attributable number or attributable fraction or disability-adjusted life years, as shown in our recently published literature on the burden of disease attributable to temperature (Cheng et al., 2019).

In the revised manuscript, we rephrased this sentence by clarifying the dengue burden and citing relevant references (lines 342-343, page 17).

References

Cheng J, Xu Z, Bambrick H, Su H, Tong S, Hu W. 2019. Impacts of exposure to ambient temperature on burden of disease: a systematic review of epidemiological evidence. Int J Biometeorol. 63(8):1099-1115. doi: 10.1007/s00484-019-01716-y.

Fan J, Wei W, Bai Z, Fan C, Li S, Liu Q, et al. 2014. A systematic review and meta-analysis of dengue risk with temperature change. Int J Environ Res Public Health. 12(1):1-15.

Wu X, Lang L, Ma W, Song T, Kang M, He J, et al. 2018. Non-linear effects of mean temperature and relative humidity on dengue incidence in Guangzhou, China. The Science of the total environment. 628-629:766-71.

11. Maybe insert some comment about serotypes as a limitation, unless this information is available.

**Response:** Since the data on the serotypes of dengue is not available in this study, we acknowledged this limitation in the revised manuscript (lines 429-431, page 21).

12. The association of heatwaves and increased outbreaks may well reflect the impact of heatwaves on human health generally. There’s quite a lot on this in non-infectious diseases. Heatwaves make people sick and effect the immune response.

**Response:** Indeed, there are many studies investigating the association between heatwaves and a number of health outcomes such as mortality and morbidity from all cause, cardiovascular disease and respiratory disease. As you mentioned, high temperatures during the heatwave period could jeopardize human health in many ways such as the dysfunctional cardiovascular, respiratory and immune systems. In the revised manuscript, we introduced relevant findings on heatwave effects on non-infectious diseases and potential immune mechanisms that may explain the association between heatwaves and incident dengue (lines 406-410, page 20).

**Reviewer #2:**

Overall general comments

The paper is well written and addresses a re-emerging disease of growing public health concern that is greatly influenced by climatic factors. There are a few grammatical errors that the authors can address by having a third party read through for language editing. Apart from addressing the specific comments mentioned above which will clarify and improve the manuscript, I find the paper is suitable for publication in PLOS NTD.

**Response:** Thanks very much for your positive comments. The manuscript has been revised based on the comments, as outlined below. Our co-authors with English native speakers carefully checked the language of this manuscript to avoid potential grammatical errors. We believe that the revised manuscript is now easier to read and understand.

Specific comments

1. The authors state clearly how high temperatures that occur as heatwaves affect occurrence of dengue outbreaks in the following lines; “Line 350: we found that temperature was responsible for a large number of dengue cases”. Then goes on to state in line 367: Noticeably, heatwaves had a much longer delayed effect on large dengue outbreaks than small and medium outbreaks” and line 371 that These findings may suggest that not only have heatwaves delayed the timing of dengue outbreak but also increased the magnitude”. It is unclear to me (and possibly for other readers who will be interested in this work) how the delay is conceived. What would have been the expected timing of the outbreaks following a heat wave? If increased magnitude of outbreak is bad as would be expected, is increased delayed outbreak a good thing? How can this delay be explained in terms of disease incidence vis a vis rise in temperature and what are the implications to public health?

**Response:** The delayed effects of temperature on human health has been well documented in the literature (Guo et al., 2014; Silveira et al., 2019), mainly because people can protect themselves within a certain period of time through active and passive protection measures such as thermoregulatory responses and utilization of air conditioning. Likewise, it may take some time to observe the effects of heatwaves on dengue outbreaks, considering the time needed for the initial negative effects of heatwaves that decrease mosquito population (Jia et al., 2019), later mosquito outbreaks due to suitable weather conditions for growth and reproduction after heatwaves (Chaves et al., 2014), dengue infection among population, and build-up of dengue outbreaks. Therefore, dengue outbreaks may occur several weeks after heatwaves.

In our data analysis, we used a lag up to 20 weeks in the model to track the delayed effects of heatwaves on dengue outbreaks. We observed that the risk of dengue outbreaks increased several weeks after heatwaves, as shown in Fig. 4 and S7 Fig. Importantly, we found that heatwaves had a much longer delayed effect on large dengue outbreaks than small and medium outbreaks. This finding suggest that small and medium heatwaves are more likely to happen in the first few weeks after heatwaves. Several months after heatwaves, large dengue outbreaks may happen.

It is a bad thing to observe the dengue outbreak, regardless the timing of outbreak, because dengue outbreak will result into substantial disease burden and economic burden. The findings of this study suggest that heatwaves increased the likelihood of dengue outbreak, and the outbreak was delayed by heatwave event. Overall, when heatwaves are predicted to happen, it is necessary to take proactive measures to prevent the potential dengue outbreaks or reduce its impacts.

In the revised manuscript, we explain potential reasons for the observed delayed effects of heatwaves on dengue outbreaks (lines 368-374, pages 18-19). Potential implications of our findings to public health have also been added to the revised manuscript (lines 383-389, page 19).

References:

Chaves LF, Scott TW, Morrison AC, Takada T. 2014. Hot temperatures can force delayed mosquito outbreaks via sequential changes in Aedes aegypti demographic parameters in autocorrelated environments. Acta Trop. 129:15-24. doi: 10.1016/j.actatropica.2013.02.025.

Guo Y, Gasparrini A, Armstrong B, Li S, Tawatsupa B, Tobias A,et al., 2014. Global variation in the effects of ambient temperature on mortality: a systematic evaluation. Epidemiology. 25(6):781-9. doi: 10.1097/EDE.0000000000000165.

Jia P, Liang L, Tan X, Chen J, Chen X. 2019. Potential effects of heat waves on the population dynamics of the dengue mosquito Aedes albopictus. PLoS Negl Trop Dis. 13(7):e0007528. doi: 10.1371/journal.pntd.0007528.

Silveira IH, Oliveira BFA, Cortes TR, Junger WL. 2019. The effect of ambient temperature on cardiovascular mortality in 27 Brazilian cities. Sci Total Environ. 691:996-1004. doi: 10.1016/j.scitotenv.2019.06.493.

2. In an attempt to provide biotic explanation to the model, authors have made statements that need clarification. In line 375 “long-lasting high temperatures affect the mosquito population and in line 376 “High temperatures during heatwaves may exceed the threshold for mosquito survival and mosquito population would be greatly reduced”. How would this result in increased cases or outbreaks? It sounds like it would have the opposite effect. In real life scenario, increased temperature can affect mosquito population negatively if the relative humidity is low. However, in high humidity, mosquito survival would not be much affected, but the high temperature would increase the virus development time (extrinsic incubation period, EIP) in the mosquito hence increasing the transmission rate. This can be considered in explaining the effect of temperature on transmission hence increased outbreaks.
**Response:** Thanks for your suggestions. High temperatures can have negative effects on mosquito population, as reported in a recent paper (Jia et al., 2019). However, the delayed effects of high temperatures on mosquito population have been less investigated (Jia et al., 2019), and little is known about how the mosquito population will change after high temperature days. It has been found that high temperature can force the delayed mosquito breeding (Chaves et al., 2014), which likely increase the risk of later dengue outbreak.

As you suggested, it is also possible that high humidity with high temperature is favourable for the development of dengue virus (reduced extrinsic incubation period), which may be another reason for the increased risk of dengue outbreak associated with heatwaves. This potential mechanism has been added to the revised manuscript (lines 397-400, page 20).

References:

Chaves LF, Scott TW, Morrison AC, Takada T. 2014. Hot temperatures can force delayed mosquito outbreaks via sequential changes in Aedes aegypti demographic parameters in autocorrelated environments. Acta Trop. 129:15-24. doi: 10.1016/j.actatropica.2013.02.025.

Jia P, Liang L, Tan X, Chen J, Chen X. 2019. Potential effects of heat waves on the population dynamics of the dengue mosquito Aedes albopictus. PLoS Negl Trop Dis. 13(7):e0007528. doi: 10.1371/journal.pntd.0007528.

3. The authors should explain how these findings have relevance to dengue outbreak prevention and response.

**Response:** The implications of our findings for dengue outbreak prevention and response have been added to the revised manuscript (lines 353-359, page 18; lines 383-389, page 19).

Specifically, findings on the heatwave effects on dengue outbreaks (heatwaves had a much longer delayed effect on large dengue outbreaks than small and medium outbreaks) suggest that it is necessary to pay close attention to the local weather conditions, considering that dengue outbreaks, in particular the large outbreak, will cause substantial disease burden and economic burden. When heatwaves are predicted to happen, proactive measures should be taken to prevent the upcoming dengue outbreaks or reduce its impacts. Because small and medium heatwaves are more likely to happen in the first few weeks after heatwaves and large dengue outbreaks may happen several months after heatwaves, different levels of early warning and response measures should be in place at different time periods to take full advantage of limited public health resources and minimize the negative consequence of heatwaves.

Findings on the dengue burden attributable to the short-term effects of temperature (temperature was responsible for a large number of dengue cases) indicate that it would be beneficial to consider the contribution of temperature for policy-makers when developing dengue burden mitigation strategies - an effective dengue forecasting system based on reliable temperature data would undeniably benefit public health. Hence, more of such efforts are required to assess the association between dengue burden and temperature so as to deepen our understanding of climate change impacts. Cost-effectiveness of developing and implementing early warning system that incorporates the short-term effects of temperature is a pressing future research area.
